# A New Case of Mitochondrial RNA Helicase SUPV3L1-Associated Neurodegenerative Disease: Ataxia, Spasticity, Optic Atrophy, and Skin Hypopigmentation (ASOASH)

**DOI:** 10.3390/genes15111406

**Published:** 2024-10-30

**Authors:** Polina Tsygankova, Denis Chistol, Tatiana Krylova, Igor Bychkov, Vyacheslav Tabakov, Tatiana Markova, Elena Dadali, Ekaterina Zakharova

**Affiliations:** Research Centre for Medical Genetics, Moscow 115552, Russia

**Keywords:** SUPV3L1, ataxia, spasticity, optic atrophy, hypopigmentation, whole genome sequencing, RNA-analysis

## Abstract

Background: The *SUPV3L1* gene encodes ATP-dependent RNA helicase SUPV3L1, which is a part of the mitochondrial degradosome complex or SUV3. SUPV3L1 unwinds secondary structures of mitochondrial RNA (mtRNA) and facilitates the degradation of mtRNA molecules. A nonsense homozygous variant in the *SUPV3L1* gene was recently associated with mitochondrial disease. Our study presents the second documented case of *SUPV3L1* pathology in humans. Methods: Whole-genome sequencing was performed on the NovaSeq 6000 platform using pair-end reading. Data analysis was performed with an in-house developed pipeline. Results: The 17-year-old female patient exhibited a diverse array of symptoms, including ataxia, spastic paraparesis, cognitive deficit, optic atrophy, and horizontal gaze-evoked nystagmus. Early onset of symptoms, such as ataxic gait and nystagmus, was noted, with subsequent progression of neurological manifestations. At the time of the observation, the proband had extensive regions of hypopigmented skin patches on the body and extremities, which have progressed over time. Whole-genome sequencing revealed compound heterozygous variants in the *SUPV3L1* gene: c.272-2A>G and c.1924A>C; p.(Ser642Arg). RNA analysis demonstrated splicing changes attributable to the c.272-2A>G variant. ELISA assay showed increased Complex I content in the patient’s fibroblasts. This case underscores the phenotypic diversity associated with *SUPV3L1* mutations, emphasizing the importance of considering mitochondrial RNA helicase dysfunction in the differential diagnosis of neurodegenerative disorders. Further elucidation of the molecular mechanisms underlying SUPV3L1-associated pathology may provide valuable insights into targeted therapeutic interventions.

## 1. Introduction

The *SUPV3L1* gene, also known as the suppressor of var1, 3-like 1 gene, is a nuclear-encoded mitochondrial RNA helicase that plays a crucial role in mitochondrial function and maintenance. While its function is not fully elucidated, emerging research suggests its involvement in various cellular processes, including mitochondrial RNA metabolism, ribosome biogenesis, and cellular stress responses.

SUPV3L1 is primarily associated with the mitochondrial RNA degradation process [1]. It functions as an RNA helicase, unwinding RNA secondary structures and facilitating the degradation of mitochondrial RNA molecules [2,3]. This activity is vital for maintaining mitochondrial RNA homeostasis, ensuring proper mitochondrial function and cellular energy production [4].

Van Esveld et al. [5] reported a pathogenic *SUPV3L1* mutation in two siblings affected with a previously undescribed neurodegenerative syndrome. The identified mutation led to a mitochondrial RNA processing defect, emphasizing the critical role of SUPV3L1 in maintaining mitochondrial RNA homeostasis. 

Understanding the variation in *SUPV3L1* genetic defects and their impact on mitochondrial function is crucial for unraveling the molecular basis of these diseases.

In our manuscript, we present clinical descriptions and molecular data on a novel case of SUPV3L1-associated recessive disorder.

## 2. Materials and Methods

### 2.1. Molecular Genetic Methods

Genomic DNA was extracted from blood samples using a QiaAMP DNA-mini kit (Qiagen, Germantown, MD, USA), following the manufacturer’s protocol.

Sanger sequencing was performed using an ABI Prism 3500XL (Thermo Fisher Scientific, Waltham, MA, USA), following the manufacturer’s protocol.

Whole-genome sequencing of the patient’s DNA was performed using the TruSeq DNA PCR-Free sample preparation kit on NovaSeq 6000 (Illumina, San Diego, CA, USA) with a mean coverage of 45X.

Bioinformatic pipeline: Sequence reads were aligned to the human reference genome hg19 using the Burrows–Wheeler Aligner (http://bio-bwa.sourceforge.net/, accessed on 1 October 2024). Single-nucleotide variants and small insertions and deletions (indels) were called with the Strelka2 Small Variant Caller (https://github.com/Illumina/strelka, accessed on 1 October 2024) and the Genome Analysis Toolkit v.4 (https://gatk.broadinstitute.org/, accessed on 1 October 2024). Structural variants were identified using Manta [https://github.com/Illumina/manta, accessed on 1 October 2024] and Delly (https://github.com/dellytools/delly, accessed on 1 October 2024). The IGV browser (https://www.igv.org/, accessed on 1 October 2024) was used to visualize the genomic data. The reported variants were annotated with their genomic coordinates, allele frequency (gnomAD database v.2.1, http://gnomad.broadinstitute.org, accessed on 26 September 2024), functional consequence, and impact level on the gene product using SnpEff v5 (http://pcingola.github.io/SnpEff, accessed on 26 September 2024). Variants were prioritized by a consensus score from a set of bioinformatic tools that predict the pathogenicity of the variant and its deleterious effect on protein (SIFT, SIFT4G, Polyphen2, MutationAssessor, FATHMM, PROVEAN, DEOGEN2, LRT, PrimateAI, MetaSVM, MetaLR, SpliceAI, MMsplice, SPiP, Spidex). Data analysis was performed using an in-home web-based NGSData-Genome interface. All variants identified by massive parallel sequencing were validated by Sanger sequencing in the proband and both parents. Variants were named according to HGVS nomenclature using the *SUPV3L1* reference sequence NM_003171.5 and the GRCh37.p13 (hg19) genome assembly.

### 2.2. RNA-Analysis from Fibroblasts

Total RNA was extracted from the patient’s cultured fibroblasts using the “Total RNA purification kit” by Norgen Biotek (https://norgenbiotek.com, Thorold, ON, Norway).

### 2.3. Fibroblasts Cultures Preparation

Primary skin fibroblast cultures were obtained from inner forearm skin biopsy fragments of the patients with confirmed primary mitochondrial diseases (*n* = 4) and healthy volunteers (*n* = 3) by the fibroblast cell culture preparation procedure. Cells were grown at 37 °C to 85% confluence in the specially proliferative medium “Amniokar” (PanEco-Ltd., Moscow, Russia).

For the research, cells were subcultured in ordinary growth medium DMEM, supplemented with 10% FBS and 200 µM uridine. All culture and sample preparation services were provided by the Common Use Center “Biobank” (Research Centre for Medical Genetics, Moscow, Russia).

### 2.4. Complex I Content in Fibroblast Lysates

Measurement of complex I content was performed using the Abcam kit, “Human NADH Dehydrogenase ELISA Kit (Complex I)” (https://www.abcam.com, Waltham, MA, USA), following the manufacturer’s instructions. Fibroblasts from 25 cm^2^ flasks were harvested using 0.25% trypsin and centrifuged at 1500 rpm for 5 min. Samples with an equal amount of extracts (lysates at 20 × 106 cells/mL) from cell pellets of the patient, patients with confirmed PMD diagnosis, healthy controls, and standards were incubated with 50 μL of antibody cocktail for 1 h.

## 3. Results

### 3.1. Clinical Case

The patient is a 17-year-old female with complaints of gait disturbance, slurred speech, choking while swallowing, excessive salivation, and vertigo.

### 3.2. Anamnesis

#### 3.2.1. 0–7 Years

The patient was born from a pregnancy threatened with termination at 20 weeks and was delivered at 40 weeks via emergency cesarean section due to uterine bleeding. Her birth weight was 3500 g, length 52 cm, and she received an Apgar score of 6/7 points. Following resuscitation, she exhibited audible cries.

During the first 6 months, her motor and pre-speech development progressed in accordance with age expectations. However, at 6 months old, following an episode of moderate pyelonephritis, her motor development decelerated. She achieved independent sitting at 10 months and began walking independently with a wide base at 18 months, albeit unsteadily. Phrasal speech emerged at 2.5 years of age, although dysarthria was noted.

At the age of 1 year, she was diagnosed with nystagmus, and at 4 years old, optic disc atrophy was confirmed. By 2.5 years old, the emergence of lower spastic paraparesis and ataxia led to a diagnosis of cerebral palsy.

At the age of 7, she started attending a specialized school for children with cerebral palsy. However, she struggled to adapt to the program due to impaired fine motor movements in her hands. Consequently, she transitioned to homeschooling.

#### 3.2.2. 7–12 Years

At 7.5 years of age, the patient’s condition deteriorated significantly. Coordination disorders and nystagmus, which became more pronounced, increased notably, accompanied by a regression in intellectual development and intensified headaches. An MRI of the brain revealed cerebellar atrophy. Upon examination at this age, the child exhibited a spastic-ataxic gait and tendon hyperreflexia in the legs, with the presence of pathological foot signs and foot clonus, along with dysarthria. A tentative diagnosis of one of the variants of spinocerebellar ataxia was considered.

Subsequent DNA analysis aimed at assessing the number of trinucleotide repeats in the *ATX1*, *ATX2*, *ATX3*, *ATX7*, *ATX8*, and *FXN* genes, responsible for various spinocerebellar ataxia variants and Friedreich’s ataxia, revealed a normal number of repeats. Additionally, no common pathogenic variants were identified in mitochondrial DNA. The lactate level was measured at 1.2 mmol/L (ref. < 2.1 mmol/L).

Upon re-evaluation of the patient at 12 years of age, the emergence of multiple hyperpigmented spots on the skin of the left leg was noted, previously reported by the mother to have been red in color. As a result, a diagnosis of hypomelanosis of Ito was established following an analysis of skin fibroblasts, which did not reveal any abnormal mosaic clone of cells. The karyotype was normal female- 46,XX.

To further elucidate the etiology of the disease, the clinical exome was sequenced, identifying a previously undescribed heterozygous variant in the nucleotide sequence of exon 33 of the *SPTBN2* gene chr11:66455365C>T. This variant resulted in the substitution p.Glu2159Lys, classified as a variant of uncertain significance (VUS). Heterozygous pathogenic variants in this gene are associated with spinocerebellar ataxia type 5. However, Sanger sequencing revealed this variant in the healthy mother of the proband, suggesting it is benign.

#### 3.2.3. Evaluation at 17 Years Old

**Figure 1:**
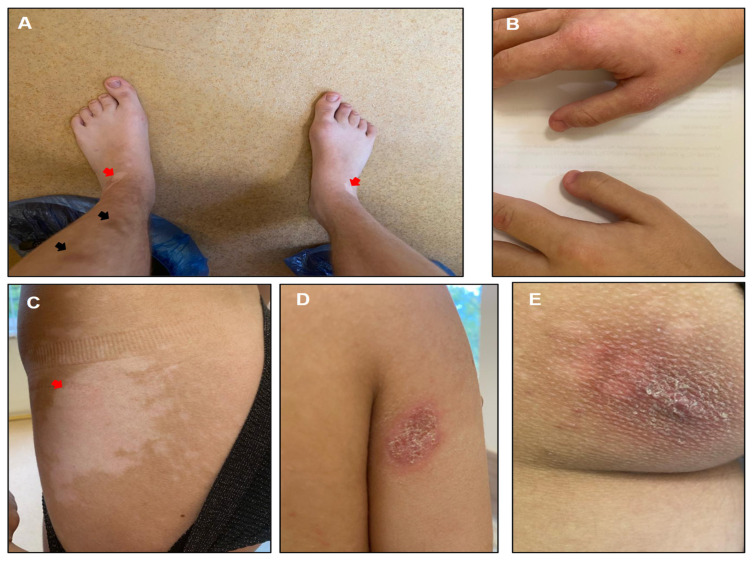
Various skin pigmentation abnormalities, including multiple depigmented spots on the legs ((**A**), red arrows) and abdomen ((**C**), red arrows), as well as hyperpigmented spots of varying sizes on the left lower leg ((**A**), black arrows). (**A**) Shortening of distal phalanges of 2–5 toes. (**B**) Shortening of distal phalanges of thumbs. (**D**,**E**) Ichthyosis-like areas of skin peeling on the right arm (**D**) and left buttock (**E**).

At 17 years of age, the patient measured 159.5 cm in height, 46 kg in weight, and had a head circumference of 53.5 cm. The examination revealed various skin pigmentation abnormalities, including multiple depigmented spots on the legs and abdomen, as well as hyperpigmented spots of varying sizes on the left lower leg and abdomen, accompanied by difficult-to-treat acne. Additionally, areas of ichthyosis-like skin patches were observed on the right shoulder and left buttock (Figure 1).

In the neurological assessment, the patient exhibited spastic tetraparesis, more pronounced in the legs, along with tendon hyperreflexia in the arms and legs and clonus of the feet. Increased muscle tone was noted in the muscles of the arms and legs. Instability in the Romberg position, intention tremor of the hands, horizontal nystagmus, and strabismus were also observed. Signs of pseudobulbar paresis and dysarthria were present. Muscle strength in both the distal and proximal muscles of the upper and lower extremities was assessed at 5 points. However, there was a decrease in both superficial and deep sensitivity in the distal regions of the arms and legs. Additionally, an intellectual deficit was noted.

**Figure 2:**
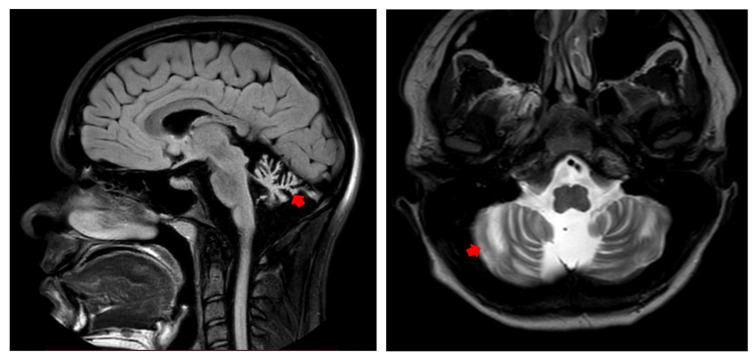
Brain MRI showing severe atrophic changes in the white matter of the cerebellum, primarily in the vermis (red arrows).

Dynamic MRI of the brain revealed atrophic changes in white matter, primarily in the vermis region of the cerebellum, with no other detectable changes in brain structures (Figure 2). An ophthalmological examination did not reveal significant pathology of the retina, cornea, or optic nerve. However, slight abnormalities, such as a border defect of the retinal nerve fiber layer and mild hypoplasia of the macula and optic nerve head, were observed.

### 3.3. Molecular Findings

#### Massive Parallel Sequencing

After obtaining negative results from whole-exome sequencing (WES) and whole-genome sequencing (WGS) conducted by commercial laboratories, we undertook a third attempt at the WGS raw data analysis. This analysis was carried out by clinical interpreters and bioinformaticians highly specialized in mitochondrial disorders at the Laboratory of Inherited Metabolic Diseases, Research Centre for Medical Genetics, Moscow, Russia.

During the repeated WGS trio analysis (proband plus parents), we identified two compound heterozygous variants in the *SUPV3L1* gene: c.272-2A>G in intron 1 and c.1924A>C; p.(Ser642Arg) in exon 14.

**Figure 3:**
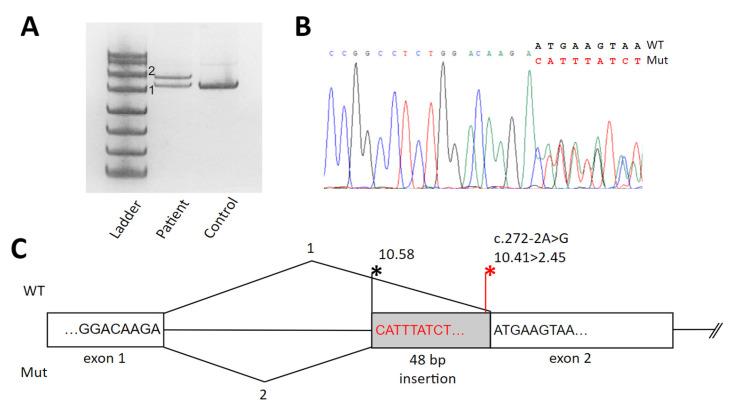
The results of cDNA analysis and a schematic illustration of splicing defects caused by the c.272-2A>G variant in the fibroblasts of the proband. (**A**) Electrophoresis of cDNA PCR fragments from the patient and control. Two bands in the patient’s sample correspond to the normal (1) and the mutated isoform with the 48 bp insertion (2). (**B**) Sanger sequencing results of the patient’s cDNA fragment. (**C**) Schematic representation of the splicing alteration caused by the c.272-2A>G variant. Asterisks indicate splice sites and their strength, calculated using MaxEntScan [6].

The c.272-2A>G variant was not reported earlier and is located within the acceptor splice site of exon 2. To evaluate the effect of this variant on splicing, we analyzed the RNA extracted from the patient’s fibroblasts. Analysis of PCR products from the *SUPV3L1* cDNA amplification demonstrated the insertion of 48 bp of intron 2, which leads to the formation of a premature stop codon r.268_269ins48; p.Asn91Thrfs*2 (see Figure 3). According to ACMG criteria, we classified c.272-2A>G as a pathogenic variant (PM2strong, PP3strong, PS3strong).

The variant c.1924A>C, p.(Ser642Arg), is present in the gnomAD database (version 4.1) with a minor allele frequency of 0.000004960. The variant is located in the donor splice site of the exon 16. Routine cDNA analysis from the patient’s fibroblasts did not reveal any significant splicing alteration. The serine at position 642 is highly conserved among vertebrate species. Multiple single nucleotide variant predictors, including PolyPhen-2, MutationTaster, SIFT, PROVEAN, dbscSNV Ada, VARITY, AlphaMissense, and REVEL, suggest a high likelihood of this substitution being deleterious. According to the PDB database, the Homo sapiens protein SUV3_HUMAN (Q8IYB8) contains two principal domains: a Helicase ATP-binding domain (amino acids 194–334) and a catalytic domain SUV3_C (amino acids 353–518), although in the initial work, Minczuk M. established the SUV3_C domain to span amino acids 650–786 [7]. In the latest work, Jain M. established that the C-terminal domain (CTD), spanning amino acids 501–722, plays an important role in the dimerization of the SUV3 protein [8].

Additionally, the variant was described in a heterozygous state in a compound with single nucleotide duplication in the unpublished paper by Green et al., May 2024 [9], in a 2-year-old patient of Slavic origin with hypotonia, motor delay, and hypopigmented lesions. According to ACMG criteria, the c.1924A>C variant is presumed to be likely pathogenic (PM2, PM3, PP3, PP5). It is noteworthy that both parents were found to be heterozygous carriers for one of the variants.

### 3.4. Complex 1 Content

**Figure 4:**
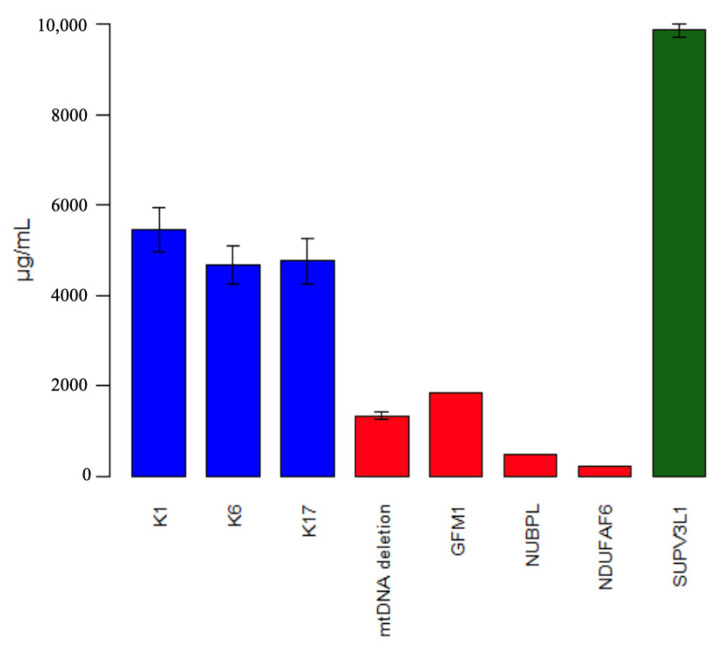
Concentration of Complex I (ELISA, Abcam) in fibroblasts. K1, K6, and K17 (blue) indicate healthy controls. Red boxes represent patients with Complex I deficiency confirmed by molecular analysis (mtDNA deletion syndrome, *GFM1*, *NUBPL*, *NDUFAF6*). *SUPV3L1* (green) corresponds to the patient described in the current publication.

To explore mitochondrial respiratory chain function, we measured Complex I content in fibroblasts. The patient’s fibroblasts exhibit almost twice the amount of Complex I content (Figure 4).

## 4. Discussion

The *SUPV3L1* gene encodes a mitochondrial RNA helicase known as SUV3, which is essential for mitochondrial RNA surveillance and degradation. This gene plays a significant role in mitochondrial RNA turnover, forming part of the mitochondrial degradosome complex, which is critical for maintaining mitochondrial function by degrading aberrant or unneeded mitochondrial RNAs and processing primary transcripts into mature mRNAs and rRNAs [1,3].

In humans, biallelic mutations in the *SUPV3L1* gene have been described by two groups [5,10], but only one family case, reported by Van Esveld et al. [5] has been published at the moment of our submission. *SUPV3L1*-associated disorders seem to exhibit a variable spectrum of presentations, including neurodevelopmental disorders, ataxia, white matter lesions, skin hypopigmentation, and visual defects. This condition is linked to the accumulation of mitochondrial double-stranded RNA (mtdsRNA), leading to dysregulation of interferon signaling and potentially activating an antiviral immune response.

Our patient exhibits a less severe form of the disease compared to the proband described by Van Esveld et al. [5]. She can walk independently, though occasionally requires support, and does not exhibit photophobia. In an unpublished manuscript by Green et al. [10], clinical and molecular data on 18 patients with *SUPV3L1*-associated disease were documented. The manifestations in these cases typically appeared before the age of 12 months. In contrast, our patient first exhibited symptoms at the age of 1 year and 8 months, presenting with an unsteady gait. All patients exhibited neurological symptoms, including delays in motor and psychomotor development and spasticity (16/18 patients), as well as microcephaly (15/18 patients). In our case, the patient was normocephalic. Interestingly, MRI showed abnormal myelination in 7 out of 8 cases, and 5 out of 7 cases showed cerebellar atrophy, which is also compatible with our case.

Additionally, 8 out of the 18 patients had areas of skin hypopigmentation. In most cases, a mild progressive course of the disease, similar to our patient, was described.

ELISA analysis of the patient’s fibroblasts showed a 2-fold increase in Complex I content. Increased Complex I nuclear subunits content could result from several underlying mechanisms, including compensatory response to mitochondrial dysfunction, where the cell tries to increase the assembly of functional respiratory chain complexes despite underlying defects, or the activation of the unfolded protein response (UPRmt). This signaling pathway upregulates the expression of mitochondrial chaperones and other proteins, potentially including Complex I subunits. 

Additionally, we revealed normal mtDNA copy numbers in the fibroblasts of our patient—a similar result to that observed in the first described patient, Van Esveld et al. [5].

In mouse models, the deletion of Supv3L1 has been shown to lead to embryonic lethality, indicating its essential role in development. Mice with a conditional knockout of Supv3L1 display premature aging phenotypes, including sarcopenia, loss of adipose tissue, and skin abnormalities, underscoring its role in maintaining mitochondrial function and overall cellular homeostasis [10]. Interestingly, mice exhibited severe skin abnormalities manifesting as ichthyosis, thickening of the epidermis, and atrophy of the dermis and subcutaneous tissue [10], which resemble the skin presentations in our case and most patients described earlier [5,10].

Research in mice also suggests that SUPV3L1 is involved in preventing the accumulation of mitochondrial double-stranded RNA, which could otherwise trigger immune responses that contribute to pathology. The interplay between mitochondrial RNA processing defects and immune activation may explain some of the disease phenotypes observed in humans and mice [5,9,10]. Overall, *SUPV3L1* is crucial for mitochondrial health, and its dysfunction can lead to severe developmental and metabolic issues in both humans and model organisms like mice.

## 5. Conclusions

This study contributes to the growing body of evidence linking pathogenic variants in the *SUPV3L1* gene to a spectrum of mitochondrial disorders. Our findings highlight the critical role of a second opinion and in-depth whole genome data analysis in undiagnosed mitochondrial disease cases, underscored by the identification of two compound heterozygous variants in the *SUPV3L1* gene in a patient with a unique clinical presentation. The patient’s phenotype, characterized by neurological symptoms such as ataxia, spasticity, as well as optic atrophy, and skin abnormalities, aligns with previous reports but also extends the clinical variability of *SUPV3L1*-associated disorders.

This case reinforces the importance of considering *SUPV3L1* mutations in the differential diagnosis of mitochondrial diseases, particularly those presenting with neurodevelopmental issues and skin pigmentation abnormalities. The potential link between mitochondrial RNA degradation defects and immune response activation offers a promising avenue for further research. Understanding the molecular mechanisms underlying *SUPV3L1* function may lead to targeted therapeutic strategies for affected individuals. Future studies are needed to elucidate the full spectrum of *SUPV3L1*-associated phenotypes and to explore the potential for personalized medicine approaches in managing these complex disorders.

## Data Availability

The original contributions presented in this study are included in the article. Further inquiries can be directed to the corresponding author(s).
